# Indication of high lipid content in epithelial-mesenchymal transitions of breast tissues

**DOI:** 10.1038/s41598-021-81426-x

**Published:** 2021-02-05

**Authors:** Siti Norbaini Sabtu, S. F. Abdul Sani, L. M. Looi, S. F. Chiew, Dharini Pathmanathan, D. A. Bradley, Z. Osman

**Affiliations:** 1 Department of Physics, Faculty of Science, University of Malaya, 50603 Kuala Lumpur, Malaysia; 2 Department of Pathology, Faculty of Medicine, University of Malaya, 50603 Kuala Lumpur, Malaysia; 3 Institute of Mathematical Sciences, Faculty of Science, University of Malaya, 50603 Kuala Lumpur, Malaysia; 4 Centre for Biomedical Physics, Sunway University, Jalan Universiti, 46150 Petaling Jaya, Malaysia; 5 Department of Physics, University of Surrey, Guildford, GU2 7XH UK

**Keywords:** Biophysics, Molecular biophysics, Biological physics, Optical physics

## Abstract

The epithelial-mesenchymal transition (EMT) is a crucial process in cancer progression and metastasis. Study of metabolic changes during the EMT process is important in seeking to understand the biochemical changes associated with cancer progression, not least in scoping for therapeutic strategies aimed at targeting EMT. Due to the potential for high sensitivity and specificity, Raman spectroscopy was used here to study the metabolic changes associated with EMT in human breast cancer tissue. For Raman spectroscopy measurements, tissue from 23 patients were collected, comprising non-lesional, EMT and non-EMT formalin-fixed and paraffin embedded breast cancer samples. Analysis was made in the fingerprint Raman spectra region (600–1800 cm^−1^) best associated with cancer progression biochemical changes in lipid, protein and nucleic acids. The ANOVA test followed by the Tukey’s multiple comparisons test were conducted to see if there existed differences between non-lesional, EMT and non-EMT breast tissue for Raman spectroscopy measurements. Results revealed that significant differences were evident in terms of intensity between the non-lesional and EMT samples, as well as the EMT and non-EMT samples. Multivariate analysis involving independent component analysis, Principal component analysis and non-negative least square were used to analyse the Raman spectra data. The results show significant differences between EMT and non-EMT cancers in lipid, protein, and nucleic acids. This study demonstrated the capability of Raman spectroscopy supported by multivariate analysis in analysing metabolic changes in EMT breast cancer tissue.

## Introduction

Breast cancers, one of the most prevalent forms of primary malignancy in females, represent a major threat to the health and longevity of women worldwide^1,2^. The majority of deaths among breast cancer patients are due to invasion and metastasis, features related to a process known as the epithelial-mesenchymal transition (EMT)^3,4^. During the EMT process, epithelial cells lose apical-basal polarity, modulate their cytoskeleton and show reduced cell–cell adhesive properties, promoting migratory capacity through the basement membrane and into connective tissue, with associated metastatic spread and elevated resistance to apoptosis^5–7^. In particular, EMT is associated with reduced expression of the epithelial marker E-cadherin (cadherin signifying calcium-dependent adhesion of a class of type-1 trans-membrane proteins) and enrichment of transcription factors, Snail, Twist, Slug and Zeb1, mRNA, important repressors of E-cadherin, as well as mesenchymal markers such as N-cadherin, vimentin and fibronectin.

Verifying biomarkers related to the EMT process are expected to be highly important in targeting EMT-associated breast cancer progression, also in developing treatment effective in inhibiting metastasis. Notwithstanding, the considerable diagnostic efficacy of current technologies, including clinical breast examination, screening mammography and tissue sampling (e.g. fine needle aspiration, core biopsy, and surgical excisional biopsy), the EMT process in cancer progression remains obscure, metastatic cancer involving multistep processes. In so doing, it is hoped to identify new targets for the prevention of metastasis and ultimately to improve breast cancer patient survival rates. Acknowledging that the onset of many diseases is preceded by biochemical change^8^, metabolomic diagnostic techniques are now used to study, profile and fingerprint the metabolites involved in the cellular processes.

Metabolomics is a fast growing field of research in terms of transcriptomics and proteomics, involving multicomponent analysis of all metabolites in cells, tissues, organisms and biological fluid^9–13^. The elucidation of biochemical pathways via investigation of metabolite levels has long depended on the use of Mass Spectrometry (MS)^14–16^ and Nuclear Magnetic Resonance (NMR)^17–19^. However, while MS offers greater sensitivity in detection of metabolites, major limitations include greater expense in sample preparation and low analytical reproducibility^20^. Furthermore, the derivative processes used to improve analytical capabilities can also result in metabolite degradation. Moreover, with metabolomics often dealing with distinguishing features between two different biological states, there is additional need for improvement in the analysis of target compounds in difficult biological matrices. Such factors notwithstanding, the complexity and high dynamic range of metabolite concentrations pose additional challenges to the efficacy of qualitative and quantitative analyses.

Sensing of the gross biochemical perturbations that are occurring in pathological conditions can be achieved through optical spectroscopic methods in unison with multivariate statistical tools data analysis, with changes reflected in the optical properties, including absorption, scattering and fluorescence^21,22^. Recent years have witnessed the increasingly popular use of Raman spectroscopy, identifying unknown components in materials and biological samples, further probing primary, secondary, tertiary, and quaternary structures of large biological molecules^23^. In respect of medical diagnostics, several advantageous features are on offer, including high chemical specificity and an ability to obtain molecular information in the absence of staining or labelling. The acquisition of spectra can also be performed in vitro, ex vivo, or in vivo, in each case avoiding disruption of the cellular environment. This is a major advantage given that most biological assays utilise chemical biomarkers, often requiring conditions non-native to the biological environment. Detailed characterizations are obtained through molecular-specific energy shifts and relative intensities of inelastically scattered light, present interests concerning associations with disease progression, with molecular changes reflected in individual bands in the Raman spectra^24^. Although Raman spectroscopy has not yet been established in the clinic, this technique has shown great potential in characterizing and discriminating between non-cancerous and cancerous tissues^25–28^, also between different pathologic grades of breast and other epithelial cancers^29–31^. The use of Raman spectroscopy in breast cancer investigations over the past 10 years and more, including in study of EMT, has been evaluated most predominantly using cell lines and/or animal models^32–39^. The corresponding Raman peaks have been the subject of a comprehensive review by Sabtu et al.^23^. Herein, for a range of *ex-vivo* human breast tissue specimens, the Raman spectroscopic model has been used to characterise the chemical morphological composition in corresponding macromolecules^40–43^, also to predict the breast tissue disease state during EMT development. In particular, attention is paid to the changes in lipid, protein and nucleic acid since these are the major structures responsible for biochemical changes in the process of cancer development^24^.

## Materials and method

### Sample preparation

The present study investigates non-lesional, EMT and non-EMT human breast cancer tissues, from 23 patients. These tissues were acquired following fully informed consent from patients who were subject to surgical treatment, with approval for use in research granted by the Medical Research Ethics Committee, University Malaya Medical Centre, in accordance with the International Conference on Harmonization—Guidelines for Good Clinical Practice (ICH-GCP) and Declaration of Helsinki. The samples were categorised into two groups; the first group comprises formalin-fixed and paraffin embedded (FFPE) dewaxed samples and the second group comprises FFPE waxed samples (Table 1). The rationale for comparing dewaxed and waxed FFPE samples was to consider the extent to which wax (paraffin) impregnation of cancer tissues affected the Raman spectroscopy findings. The study was piloted with waxed samples (10 samples—Group 2) without inclusion of any non-lesional tissue. However, this was discontinued in favour of dewaxed samples (27 samples—Group 1) to eliminate the confounding effect of wax on the Raman analysis. Group 1 was the main study cohort and included non-lesional material whenever this was available for the cohort cases in the pathology archive. Although not comparable in sample size, both Group 1 and Group 2 findings are shared in this report for completeness of data. There was a duplication of four cancers (2 EMT positive and 2 EMT negative) where both waxed and dewaxed samples were analysed. These are identified in Table 1. Since the study involves human samples available in the diagnostic pathology archives, it was limited by availability of such tissues. Only 10 breast cancers (4 EMT positive and 6 EMT negative) had non-lesional tissue from the same excision (mastectomy) available in the archives. All samples were from pre-treatment patients. This is to avoid the issue of neoadjuvant chemotherapy or radiotherapy confounding the EMT status in this study. To avoid compromising future pathological review for patient care, FFPE blocks containing scanty amounts of tumour material were excluded from the Raman study.

**Table 1:** The breast tissue samples classified on the basis of pathology and material source.

Groups	Sample number	Sample pathology		Material source
Grou9p 1	EMT-9	Breast cancer-EMT	Same patient	FFPE (dewaxed)
	EMT-10	Non-lesional		FFPE (dewaxed)
	EMT-11	Breast cancer- EMT	Same patient	FFPE (dewaxed)
	EMT-12	Non-lesional		FFPE (dewaxed)
	EMT-13	Breast cancer-EMT	Same patient	FFPE (dewaxed)
	EMT-14	Non-lesional		FFPE (dewaxed)
	EMT-15	Breast cancer-EMT	Same patient	FFPE (dewaxed)
	EMT-16	Non-lesional		FFPE (dewaxed)
	EMT-17	Breast cancer-EMT		FFPE (dewaxed)
	EMT-18	Breast cancer-Non-EMT		FFPE (dewaxed)
	EMT-19	Breast cancer-EMT		FFPE (dewaxed)
	EMT-20	Breast cancer-EMT		FFPE (dewaxed)
	EMT-21	Breast cancer-EMT		FFPE (dewaxed)
	EMT-22	Breast cancer-EMT		FFPE (dewaxed)
	EMT-23	Breast cancer-non-EMT	Same patient	FFPE (dewaxed)
	EMT-24	Non-lesional		FFPE (dewaxed)
	EMT-25	Breast cancer-non-EMT	Same patient	FFPE (dewaxed)
	EMT-26	Non-lesional		FFPE (dewaxed)
	EMT-27	Breast cancer-non-EMT	Same patient	FFPE (dewaxed)
	EMT-28	Non-lesional		FFPE (dewaxed)
	EMT-29	Breast cancer-non-EMT	Same patient	FFPE (dewaxed)
	EMT-30	Non-lesional		FFPE (dewaxed)
	EMT-31	Breast cancer-non-EMT	Same patient	FFPE (dewaxed)
	EMT-32	Non-lesional		FFPE (dewaxed)
	EMT-33	Breast cancer-non-EMT	Same patient	FFPE (dewaxed)
	EMT-34	Non-lesional		FFPE (dewaxed)
	EMT-35	Breast cancer-non-EMT		FFPE (dewaxed)
Group 2	EMT-13	Breast cancer-EMT		FFPE (waxed)
	EMT-36			FFPE (waxed)
	EMT-37			FFPE (waxed)
	EMT-38			FFPE (waxed)
	EMT-9			FFPE (waxed)
	EMT-39	Breast cancer-non-EMT		FFPE (waxed)
	EMT-40			FFPE (waxed)
	EMT-33			FFPE (waxed)
	EMT-41			FFPE (waxed)
	EMT-18			FFPE (waxed)

The pathology of all samples entered for Raman analysis are shown in Table 1. Of these, 27 FFPE (subsequently dewaxed) samples from 17 patients (EMT-9 to EMT-35) have also been discussed by Sobri et al. 2020^44^. To minimise tumour heterogeneity, all cancers in this study were of the invasive ductal type. Lobular carcinomas were excluded as there are known to be innately negative for E-cadherin, as were sarcomas (which are innately positive for vimentin) and preinvasive cancers. In addition, all cancers in this study had been routinely assessed for molecular predictive classification using immunohistochemistry (Ventana BenchMark automated system) for estrogen receptor (ER), progesterone receptor (PR) and HER2 according the American Society of Clinical Oncology/College of American Pathologists (ASCO/CAP) guidelines^45,46^. ER and PR positive cancers were classified as hormone receptor (HR) positive regardless of the HER2 expression. Cancers negative for ER and PR, and were HER2 positive (i.e. HER2 IHC score 3 or IHC score 2 but confirmed amplified by in-situ hybridization) were classified as HER2 enriched. Cancers negative for ER, PR and were not HER2 enriched, were classified as triple negative (TN).

In regard to sampling, fresh mastectomy or tumour excision specimens of breast cancer, received by the histopathology laboratory, have been sliced to some 1 cm thickness, subsequently immersed in 10% neutral buffered formalin^*^ to allow tissue fixation (preservation). The ratio of volume of formalin to tissue was about 10:1 while the duration of fixation was a minimum of 6 h, larger specimens (such as mastectomies) typically being fixed overnight. After fixation, the specimens were sampled for tumour, surgical margins and other areas of interest. Non-lesional samples were from mastectomies performed for removal of tumour and were not separate biopsies. The sample would accordingly not be immediately adjacent to the tumour and was usually several cm away as the purpose of such a sample was to examine the normal portion of the breast.

Each sample, of some 3 to 5 mm in thickness and not more than 2 cm × 2.5 cm in length and breadth, was placed into a uniquely labelled plastic cassette and kept in 10% neutral buffered formalin to await subsequent processing into formalin-fixed paraffin embedded (FFPE) blocks.

The processing of the samples, from formalin-fixed to a paraffinised block was carried out sequentially, with dehydration by alcohol followed by use of xylene for clearing of alcohol and subsequent replacement of xylene with paraffin wax (via impregnation). Conducted in the Department of Pathology, University of Malaya, this process was completely automated using a Leica TP 1020 tissue processor. The processing schedule was as follows:

1. Two changes of 10% formalin for 81 min each.
2. Two changes of 95% alcohol for 81 min each.
3. Three changes of 100% alcohol for 81 min each.
4. Two changes of xylene for 81 min each.
5. Three changes of wax for 81 min each.

After impregnation with paraffin, the tissue was embedded in a desired orientation in the cassette with a metal mould as the backing upon a hot plate. The cassette was then filled with liquid paraffin and placed on a cold plate to solidify the paraffin. The paraffin block was then popped out of the mould, creating a formalin fixed paraffin embedded (FFPE) block with the plastic cassette which was then ready for microtome sectioning. Microtomed sections were used for histopathology examination^**^. The method applied here has been described by Sobri et al. 2020^44^.

^*^10% neutral buffered formalin was manually prepared from monobasic Sodium phosphate 175 g, dibasic Sodium phosphate 818.5 g, formaldehyde 37% (5 L and water 45 L), with pH of 6.5. Hence the actual amount of dissolved formaldehyde in the 10% formalin was 3.7–4.0%.

^**^Histological diagnosis of breast carcinoma—In the routine histopathology laboratory of the Department of Pathology, University of Malaya, 4 micron-thick sections were microtomed from the FFPE, dewaxed and stained with Haematoxylin and Eosin (H&E) for examination under the microscope for histopathology diagnosis.

### Determination of EMT status by immunohistochemistry (IHC) and scoring

**Figure 1:**
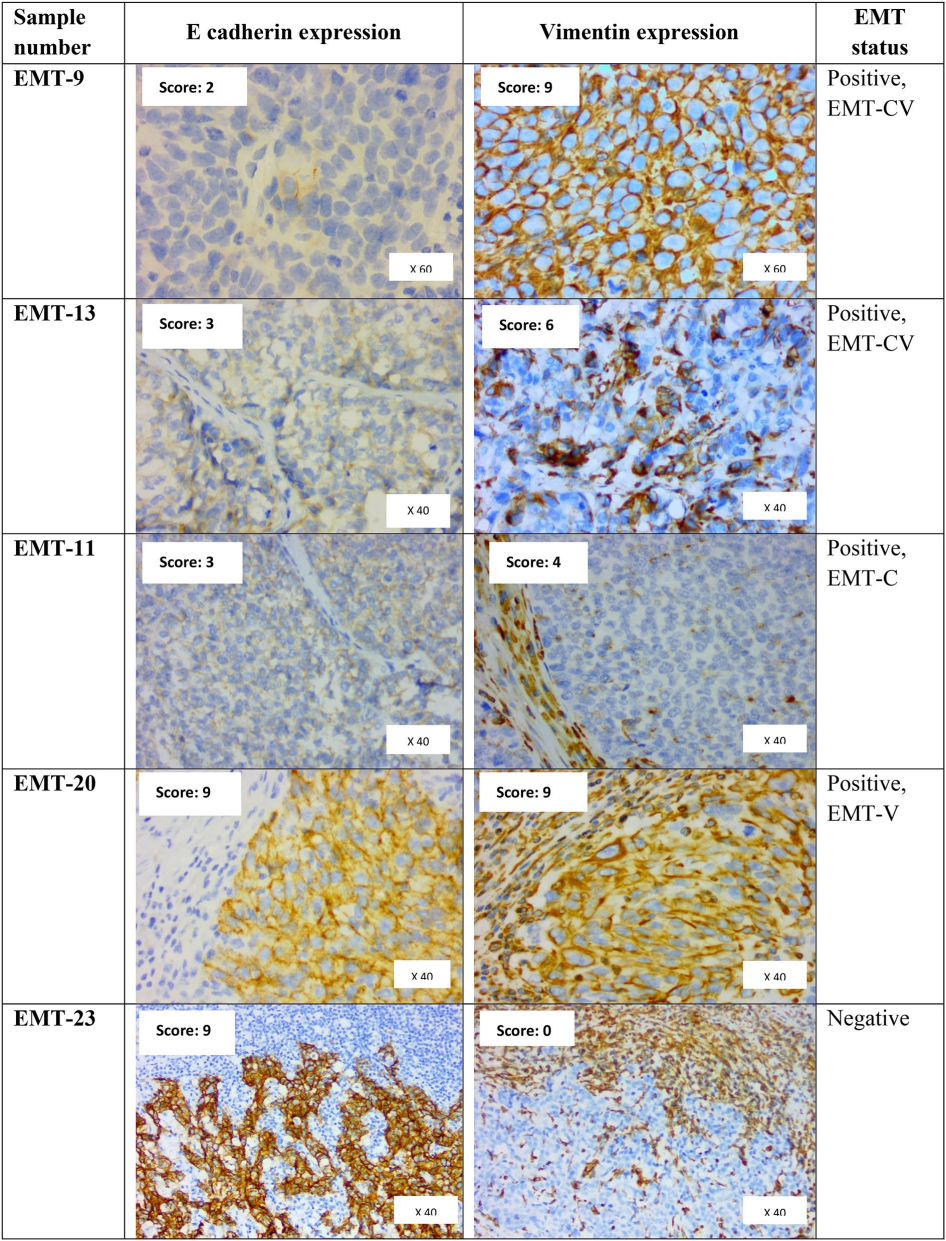
Photomicrographs of five breast cancer samples illustrating their E-cadherin and Vimentin scores, captured at comparable magnifications. Positive IHC expression is depicted by brown colour staining of the cells. Images of samples EMT-11, EMT-20 and EMT-23 illustrate the strong Vimentin staining of stromal cells which are not considered for scoring of cancer cells. EMT-20 illustrates the difference in morphology of vimentin-positive tumour cells at the right lower triangle of the picture (which show the cancer characteristics of cellular pleomorphism and enlarged nuclei) from vimentin-positive stromal (benign) cells (which are elongated and have small bland nuclei). The column at the right indicates the designated EMT status of the illustrated samples.

For this study, 12 breast cancers exhibiting EMT and 11 breast cancers not exhibiting EMT were selected from a concurrent study on EMT prevalence in human breast cancer tissues, archived at the Department of Pathology, University of Malaya. In brief, 4 micron-thick sections microtomed from FFPE blocks of breast cancers were run on a Ventana Benchmark XT automated system for IHC staining using a mouse-monoclonal antibody to E-cadherin (1:50; Dako: Clone NCH-38) and Vimentin (1:500 Dako:Clone V9). Positive IHC expression is depicted by brown colour staining of the cells. As is standard practice in the UMMC laboratory, a positive control is incorporated with each test slide. The positive control for E-cadherin is a known E-cadherin positive breast cancer while normal breast epithelium serves as an internal positive control. The positive control for Vimentin is lymphoid cells in human tonsil, while smooth muscle cells of blood vessels also serve as internal positive control. Cytoplasmic membranous expressions by cancer cells for E-cadherin and Vimentin were assessed microscopically and semiquantified for: (i) percentage of malignant cells expressing E-cadherin or Vimentin, as 0 (0– < 1%), 1 (1–10%), 2 (11–50%), 3 (more than 50%) and; (ii) intensity of staining: 0 (negative), 1 (weak), 2 (moderate) and 3 (strong). For both E-cadherin and vimentin independently, the percentage and intensity scores were multiplied to obtain a final score. Final scores of 0–4 were classified as low expression and scores of 6–9 as high expression. EMT was considered to be present if the cancer showed a low E-cadherin final score and/or a high Vimentin final score. In addition, EMT positivity was further classified as “complete” (EMT-CV) when there was both a low E-cadherin and high Vimentin score and as “intermediate” with there was either a low E-cadherin or high Vimentin score. Intermediate EMT was further categorised as EMT-C where EMT positivity was based only on a low E-cadherin score and as EMT-V where EMT positivity was based only on a high Vimentin score^7^. Scoring for E-cadherin and Vimentin was achieved through consensus scoring by four practising histopathologists (including two of the authors) in a larger concurrent study on EMT, of which the current study is a subset. Only expressions in visualised cancer cells were scored and not non-cancer elements (like stromal cells, blood vessels or inflammatory cells). Figure 1 provides images of an assortment of samples illustrating these categories, of which EMT-20 also illustrates the difference in morphology between vimentin-positive cancer cells and vimentin-positive stromal (benign) cells. Table 2 details the EMT scores of the various cancers tabulated against molecular predictive status.

**Table 2:** EMT scores and molecular classification of breast cancer samples.

Sample number	Vimentin			E-cadherin			EMT status	Molecular classification (HR/HER2/TN)
	% Score	Intensity score	Final score	% Score	Intensity score	Final score		
EMT-9	3	3	9	2	1	2	Positive, EMT-CV	TN
EMT-11	2	2	4	3	1	3	Positive, EMT-C	HR
EMT-13	2	3	6	3	1	3	Positive, EMT-CV	TN
EMT-15	2	3	6	3	3	9	Positive, EMT-V	TN
EMT-17	3	3	9	3	3	9	Positive, EMT-V	TN
EMT-18	0	0	0	3	2	6	Negative	TN
EMT-19	3	3	9	3	1	3	Positive, EMT-C	TN
EMT20	3	3	9	3	3	9	Positive, EMT-V	TN
EMT-21	2	3	6	3	2	6	Positive, EMT-V	TN
EMT-22	0	0	0	3	1	3	Positive, EMT-V	HR
EMT-23	0	0	0	3	3	9	Negative	HR
EMT-25	0	0	0	3	3	9	Negative	HR
EMT-27	0	0	0	3	3	9	Negative	HR
EMT-29	0	0	0	3	3	9	Negative	HER2
EMT-31	1	3	3	3	3	9	Negative	HR
EMT-33	0	0	0	3	2	6	Negative	HER2
EMT-35	1	3	3	3	2	6	Negative	HER2
EMT-36	2	3	6	0	1	0	Positive, EMT-V	TN
EMT-37	0	0	0	2	2	4	Positive, EMT-C	HER2
EMT-38	3	3	9	3	3	9	Positive, EMT-V	HR
EMT-39	0	0	0	3	2	6	Negative	TN
EMT-40	1	1	1	3	2	6	Negative	TN
EMT-41	0	0	1	3	2	6	Negative	HR

### Raman measurement

Raman spectra of the cancerous breast tissues were acquired using a Renishaw Raman system (Apply Innovation, Gloucestershire, UK) available at the Department of Physics, University of Malaya. The spectroscopy consists of a laser, objective lens, and charge-coupled device (CCD) detector. To excite the sample, a laser excitation beam of 532 nm was focused onto the sample through a 50 × objective microscope. Each sample was exposed to the laser excitation for 20 s, detecting in the spectral range 100–3200 cm^−1^. Raman spectra were displayed through use of a computer using Renishaw Wire software. Three to five Raman spectra were acquired from each tissue sample.

In regard to phospholipids, a major constituent of the plasma membrane forming the outermost layer of animate cells, they are composed of two fatty acids that help form a diacylglycerol. The latter activates proteins that have a role in various signalling cascades. The Raman signal sensitivity of the fatty acids was described in detail in Levchenko and Qu (2018), the authors recording that the ratio of the bands at 1665 cm^−1^ (this being the stretching mode, proportional to the amount of unsaturated C=C bonds) and 1440 cm^−1^ (this being the C–H_2_ bending mode, proportional to the amount of saturated C–C bonds) being indicative of the degree of saturation of the fatty acids. In present analysis, the respective bands are observed to centre around 1659 cm^−1^ and 1441 cm^−1^, a minor variation to that reported in Levchenko and Qu (2018)^47^. For the nucleic acids, the phosphodiester backbones (O–P–O) exhibit many of the spectral complexities of DNA and RNA, having stretching motion that give rise to peaks in the 800 to 900 cm^−1^ region^48^. The nucleosides are sensitive to the torsion of the phosphodiester bond network of the nucleic acid backbone structure and do not depend on the identity of the attached nucleotic base^49^. Additionally, Raman spectroscopy can distinguish between unique backbone conformations of A-DNA (807 ± 3 cm^−1^), B-DNA (835 ± 7 cm^−1^) and Z-DNA (745 ± 2 cm^−1^).

### Data analysis

#### Pre-processing

**Figure 2:**
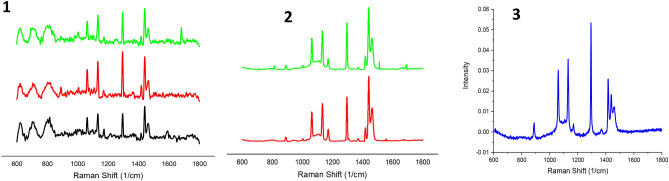
The pre-processed Raman spectra from all the samples obtained from the average of all the samples. Figure label number 1 is for FFPE dewaxed samples (Group 1) and figure label number 2 is for FFPE waxed samples (Group 2). Figure label number 3 is pure paraffin. (Black line: Non-lesional; Red line: EMT; Green line: Non-EMT).

Data pre-processing is an important step in improving the accuracy of subsequent classification analyses^50^. As the Raman spectra are prone to a variety of contaminants (detected cosmic ray events, fluorescence background, noise etc.), all Raman spectra were pre-processed using the Origin software. The eightpoint baseline correction was applied to each spectrum. Raman spectra were smoothed by using a Savitzky-Golay filter (5th order, 13 points) to reduce the noise^51^. Subsequently, the intensity of the Raman spectra were corrected by vector normalization^51^, the pre-processed data then being ready for further analysis. Example of pre-processed Raman spectra data from non-lesional, EMT and non-EMT breast tissue obtained from the average of all samples are shown in Fig. 2, with apparent appreciable paraffin contribution bands within the low wavenumber region for both dewaxed and waxed samples. These are strongly overlapped with important tissue components throughout the entire spectrum that have been obtained for each sample.

#### Statistical analysis

Multivariate analysis was performed on each pre-processed Raman spectrum, seeking differences between the various cancerous human breast tissue samples under study. Principal component analysis (PCA) is employed as a pre-processing method to reduce the number of variables (the signals obtained from a great amount of experimental data) to a value near real (in our case the number of biochemical signals detected by Raman). In this work, the first three principal components (PC) are used for Group 1 and the first five PCs for Group 2. These PCs were then used in performing independent component analysis (ICA) for paraffin extraction.

With the sample tissues embedded in paraffin, it is crucial to extract and eliminate the paraffin signals, prominent wax peaks tending to interfere with Raman spectral data^52,53^. Commonly, and as previously mentioned, the FFPE tissue samples are dewaxed using xylene. However, previous studies have shown the dewaxing procedure to be a time consuming process, with the structures of the samples potentially altered and a residual layer of paraffin potentially remaining in the sample tissues^52,54^. In this work, the Raman spectra were digitally dewaxed using ICA and Non-Negative Least Squares (NNLS) analysis as proposed by Gobinet et al. and Meksiarun et al.^53,55^. ICA is applied to estimate the paraffin component to extract pure component information from the spectra of FFPE tissue samples. In the present study, ICA was performed using the FastICA algorithm for estimation of the paraffin component in the breast cancer tissue spectra.The NNLS fittings were performed using the MATLAB software (Mathworks Inc., MATLAB version R2017b). Non-lesional, EMT and non-EMT breast tissue samples were compared in terms of Raman intensity by the analysis of variance (ANOVA) test followed by Tukey’s multiple comparison’s test. The ANOVA test was also performed to see whether dewaxing affected the Raman spectrum measurements of the three aforementioned samples.

## Results and analysis

We understand the work herein to represent the first known Raman spectroscopic study on EMT in human breast tissue, the data being supported by multivariate analysis. Previous studies by others have used cell lines in investigating molecular exchanges occurring in the EMT process^36–38^, comparisons being made between EMT and non-EMT tissue, including extraction of general metabolic features of neoplastic cells. This has led to identification of changes in nucleic acid, protein and lipid^56^, reflected in the lower wavenumber (LWN) range, 600 to 1800 cm^−1^^38^.

### Paraffin removal process

**Figure 3:**
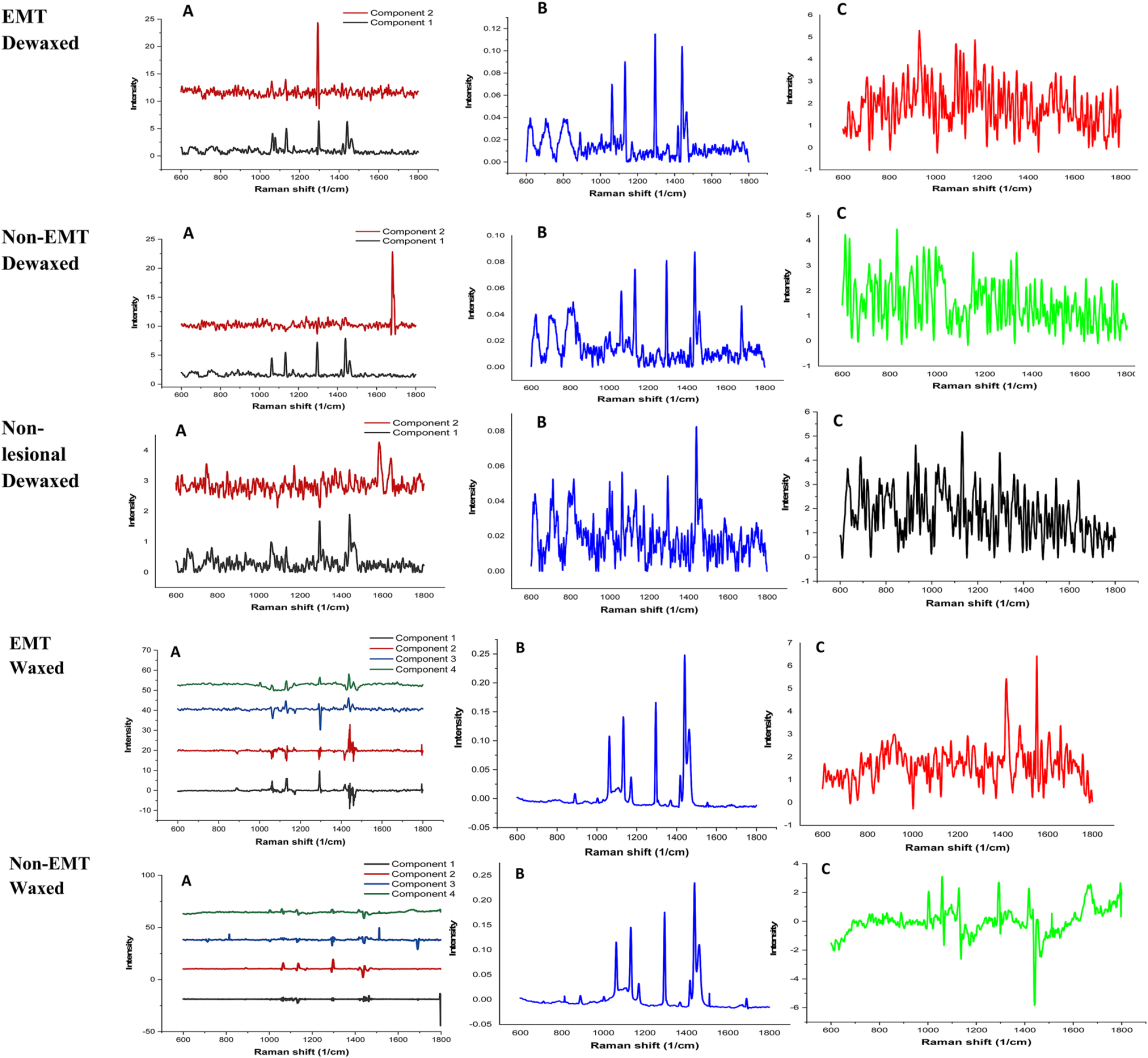
(**A**) The estimated paraffin components from ICA, (**B**) difference of before-after paraffin removal and (**C**) Raman spectra after paraffin removal.

Multivariate analysis allows delineation of the biological components of EMT in breast cancer tissues, the paraffin signal from the various Raman spectra effectively being removed. Example Raman spectra following paraffin removal within the fingerprint region (600–1800 cm^−1^), for all types of breast tissue samples, are shown in Fig. 3. From these figures, it is apparent that the EMT and non-EMT breast cancer tissues exhibit differences, albeit at a rather subtle level, in terms of intensity, position, shape and peak assignments in the Raman spectra.

The method of ICA for paraffin component extraction from FFPE cancer tissues were reported by Vrabie et al. and Meksiarun et al.^53,57^. In this study the ICA was employed by FastICA algorithm to estimate the paraffin component in the breast tissue samples as shown in Fig. 3. PCA was performed prior to ICA. The extracted paraffin components separated by ICA are shown in Fig. 3A, resembling that of pure paraffin. The estimated components later were used for subtraction by applying NNLS. In this study, we found that two components for dewaxed samples and four components for waxed samples were able to remove the paraffin bands. The residuals from Raman spectra before extracting the Raman spectra, after paraffin removal is shown in Fig. 3B. The Raman spectra after the paraffin removal process is shown in Fig. 3C.

All the prominent bands are obtained from the Raman spectra of paraffin wax extracted. We only focus on the changes of lipid, protein and nucleic acid in the samples. Firstly, we compared three breast tissues; non-lesional, EMT, and non-EMT of the FFPE dewaxed samples. Then we compared two tissues: EMT and non-EMT of FFPE of waxed samples. It should be noted again that the peaks were obtained from the average of all samples. All the assignments are based on the review by Sabtu et al.^23^.

### Lipid

#### Dewaxed samples

**Figure 4:**
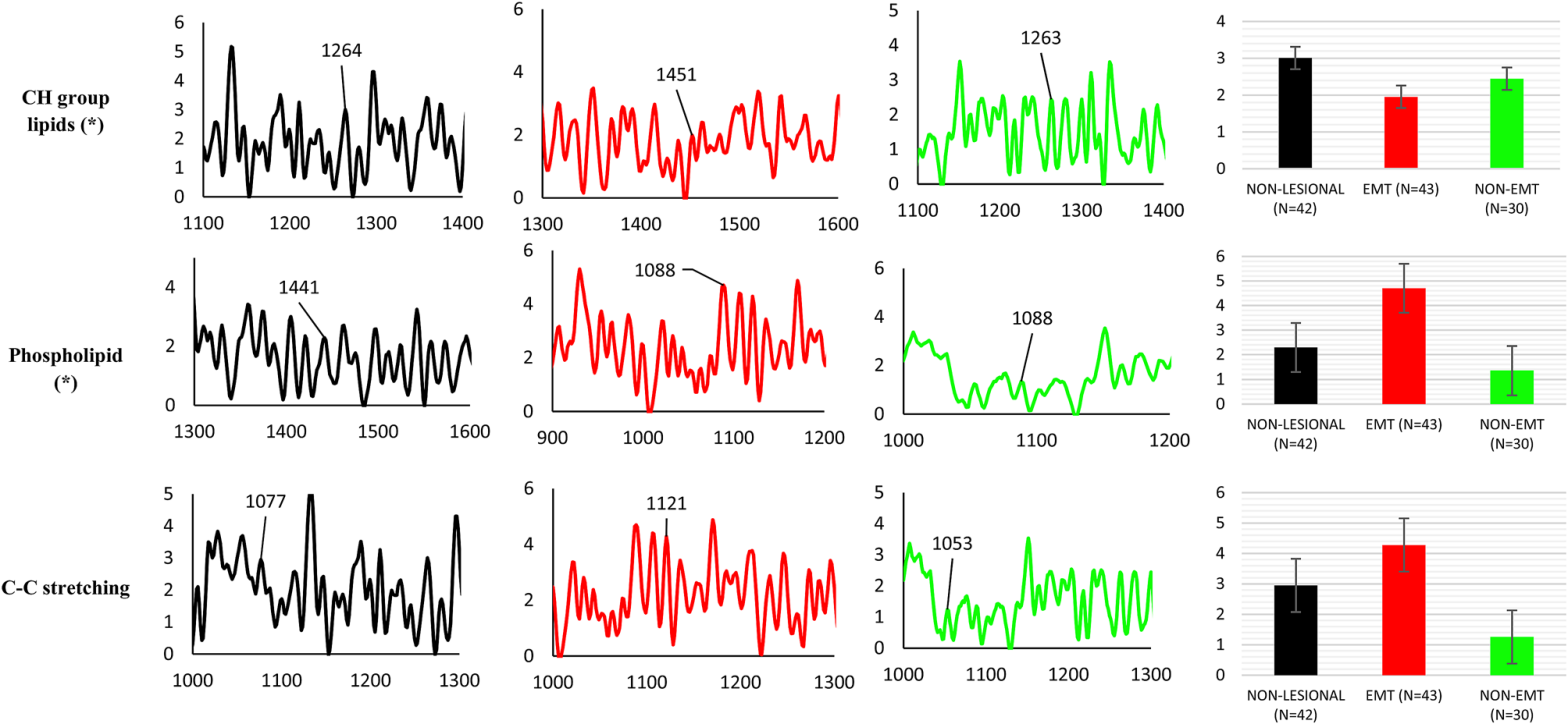
Summary of lipid vibrational bands observed for dewaxed samples (Group 1) in non-lesional, EMT and non-EMT breast cancer tissue. The x- and y-axes represent the wavenumber and normalised (vector normalisation) intensity respectively. The black colour trace represents the non-lesional tissues, red colour represents EMT tissues and green colour represents non-EMT tissues. Comparison of the relative intensity of lipid Raman bands in dewaxed samples between non-lesional, EMT and non-EMT breast tissue samples are depicted in the bar charts. Each column represents the average of spectra for each type of tissue. The value of N represents the number of Raman spectra acquired for each sample, shown within the brackets. The error bar represents standard deviation. Note: the x-axis represents the tissue type and y-axis is Raman intensity. (*) denotes a statistically significant difference between the groups reported by the ANOVA test.

**Figure 5:**
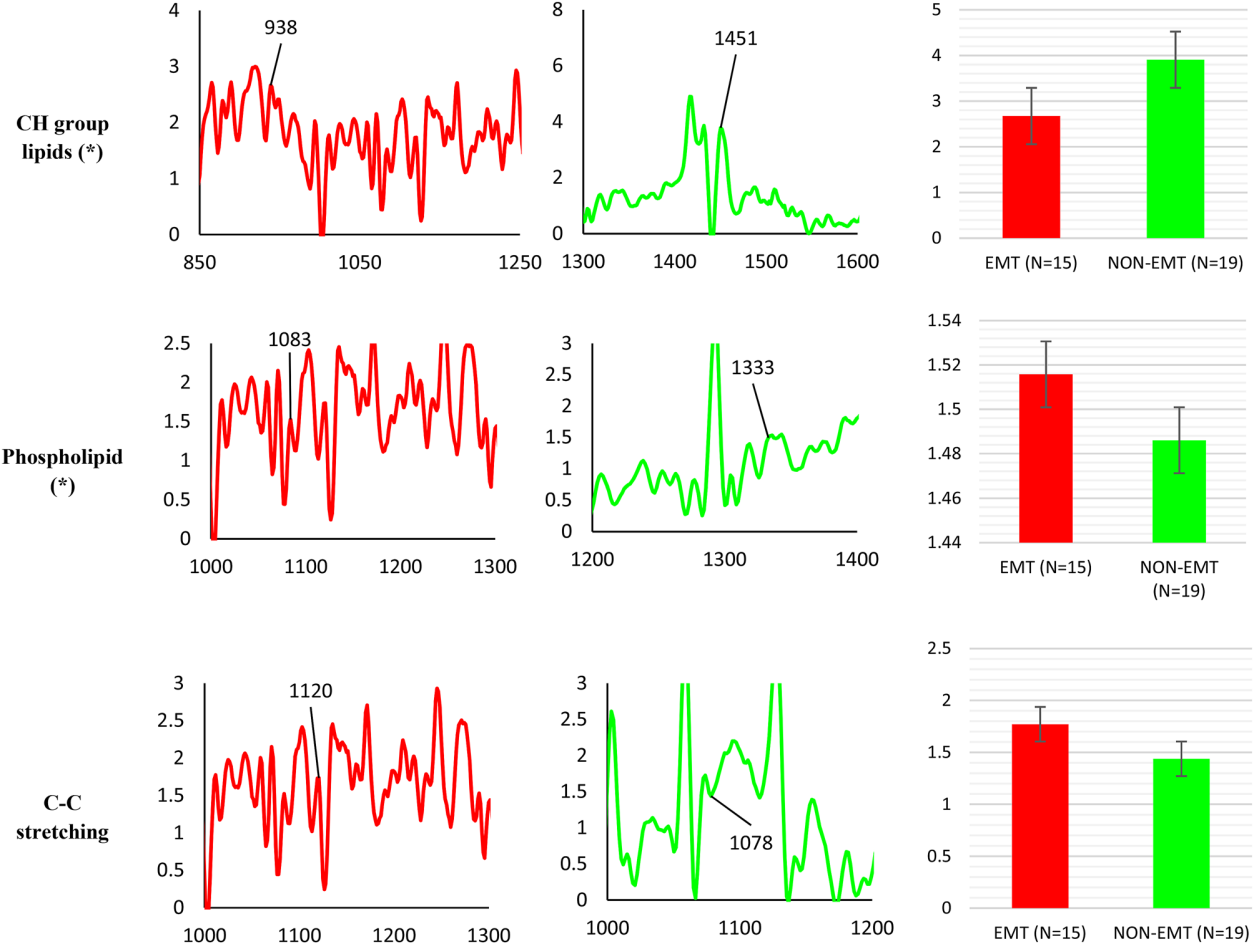
Summary of lipid vibrational bands observed for waxed samples (Group 2) in EMT and non-EMT breast cancer tissue. The x- and y-axes represents the wavenumber and normalised (vector normalisation) intensity respectively. The black colour represents the non-lesional tissues, while red represents EMT tissues and green represents non-EMT tissues. Comparison of the relative intensity of lipid Raman bands in waxed samples between EMT and non-EMT breast tissue samples is depicted in the bar charts. Each column represents the average of spectra for each type of tissue. The value of N represents the number of Raman spectra acquired for each sample, shown within the brackets. The error bar represents standard deviation. Note: the x-axis represents the tissue type and y-axis is Raman intensity. (*) denotes a statistically significant difference between the groups reported by the t-test.

Raman spectra bands consistent with the presence of lipid are found in all types of samples; non-lesional, EMT and non-EMT tissues. The prominent peak lipid bands are shown in Fig. 4 complete with peak assignments. The peaks were obtained from the average of all samples. As seen in Fig. 4, the Raman CH group signal in EMT tissues, centered around 1451 cm^-1^, are of lower intensity than that of the corresponding CH group signals in both non-lesional and non-EMT tissue, centered around 1264 cm^−1^ and 1263 cm^−1^, respectively. We found that the CH lipid bands in non-lesional tissues are 42% and 21% greater in intensity in comparison to that EMT and non-EMT tissues respectively. Phospholipid was found in non-lesional tissues at peaks of 1441 cm^−1^ and in both EMT and non-EMT tissues at peaks of 1088 cm^−1^ with greater intensity by 60% and 110% in EMT tissue samples compared to that non-lesional and non-EMT tissue respectively. At position 1121 cm^−1^ and 1053 cm^−1^, C–C stretching mode in lipid is found to exist in EMT and non-EMT tissues respectively while in non-lesional tissue the C–C stretch is found at peak 1077 cm^−1^. The greatest intensity of lipid C–C stretching was found in the EMT tissues; 109% higher intensity compared to that non-EMT tissues and 37% higher intensity compared to that non-lesional tissue. Comparison of the relative intensity of non-lesional, EMT and non-EMT tissue are also included in Fig. 5.

#### Waxed samples

The Raman spectra of lipid found in the waxed samples are depicted in Fig. 5 together with the peaks assignment. The Raman band observed at peak 938 cm^−1^ in EMT tissues and 1451 cm^−1^ in the non-EMT tissues are marker peaks of the CH group of lipid, showing 37% greater intensity in the non-EMT tissues compared to the EMT tissues. Phospholipid was found in both EMT and non-EMT tissues with slightly higher intensity in EMT tissues by 2% compared to non-EMT tissues. The C–C stretching of lipid was found at peak 1120 cm^−1^ for EMT tissues and 1078 cm^−1^ for non-EMT tissues, with greater intensity by 21% in EMT tissues compared to that of non-EMT tissues. A comparison of the relative intensity of both EMT and non-EMT tissue is shown in Fig. 5.

Numerous studies have indicated that abnormal levels of lipids in tissues correlate with metastasis of various types of cancer, including breast cancer^36,37^. An early feature of carcinogenesis is the activation of lipid metabolism, being the hallmark of many types of cancer^36,58^. It has been reported that lipids have an important role in controlling the adhesion of cell and migration processes^38,59^, a matter supported by clinical data showing lipid-rich breast cancer to be responsible for half of the deaths of patients with cancer^59,60^. With EMT a process which acquires a mesenchymal phenotype with increased migratory features, present observation of high intensities of Raman bands corresponding to lipids in EMT tissues are seen to be in accordance with this.

The results from Raman investigations of phospholipid in breast tissue obtained herein is in line with previous work by Marro et al. 2014^36^ and Chaturvedi et al. 2016^38^, both indicating the increases of phospholipid to be associated with the EMT process. Present results have shown that the phospholipid intensity in EMT tissue to be 69% greater compared to that of non-lesional tissues. For CH group lipids the signal intensity has been observed to be significantly greater in non-lesional tissues in comparison to that of EMT and non-EMT tissues, agreeing with the findings of Brozek-Pluska et al. 2012^61^ and Li et al. 2017^62^.

### Protein

#### Dewaxed samples

**Figure 6:**
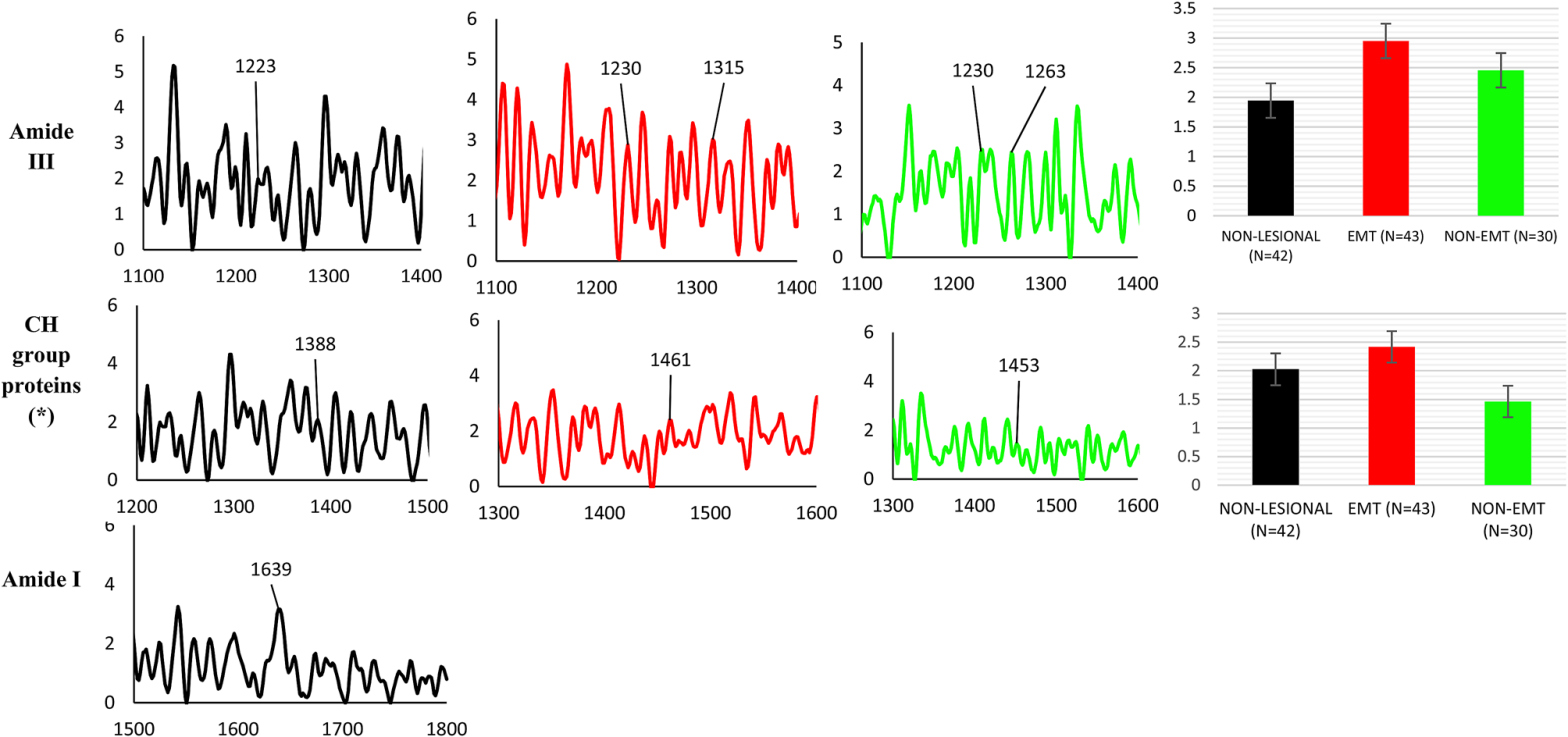
Summary of protein vibrational bands observed for dewaxed samples (Group 1) of non-lesional, EMT and non-EMT breast cancer tissue. For each case, the x-axis represents the wavenumber while the y-axis represents the intensity. The black colour represents the non-lesional tissues, while red represents EMT tissues and green represents non-EMT tissues. Comparison of relative intensity of protein Raman bands for dewaxed samples of non-lesional, EMT and non-EMT breast tissue is depicted in the bar charts. Each column represents the average of spectra for each type of tissue. The value of N represents the number of Raman spectra acquired for each sample, shown within the brackets. The error bar represents standard deviation. Note: the x-axis represents the tissue type and y-axis is Raman intensity. (*) denotes a statistically significant difference between the groups reported by the ANOVA test.

In the present work, greater intensity protein bands were observed in EMT compared to non-lesional and non-EMT breast cancer tissue. In non-lesional tissue Amide III was centered around 1223 cm^−1^ while in EMT tissue it was found at 1230 cm^−1^ and 1315 cm^−1^; in non-EMT tissues the Amide III was found at 1230 cm^−1^ and 1263 cm^−1^. CH group protein peaks were found for all three tissue types, at 1388 cm^−1^ for non-lesional tissues, 1461 cm^−1^ for EMT tissues and 1453 cm^−1^ for non-EMT tissues. The peak assigned to Amide I, a collagen assignment, was found at 1639 cm^−1^ in non-lesional tissues while it was absent in both EMT and non-EMT breast cancer tissues. Figure 6 presents mean Raman spectra for peaks attributed to proteins in non-lesional, EMT and non-EMT breast tissues samples. Comparison is made of the intensities of the protein bands in all samples, as shown in Fig. 6.

#### Waxed samples

**Figure 7:**
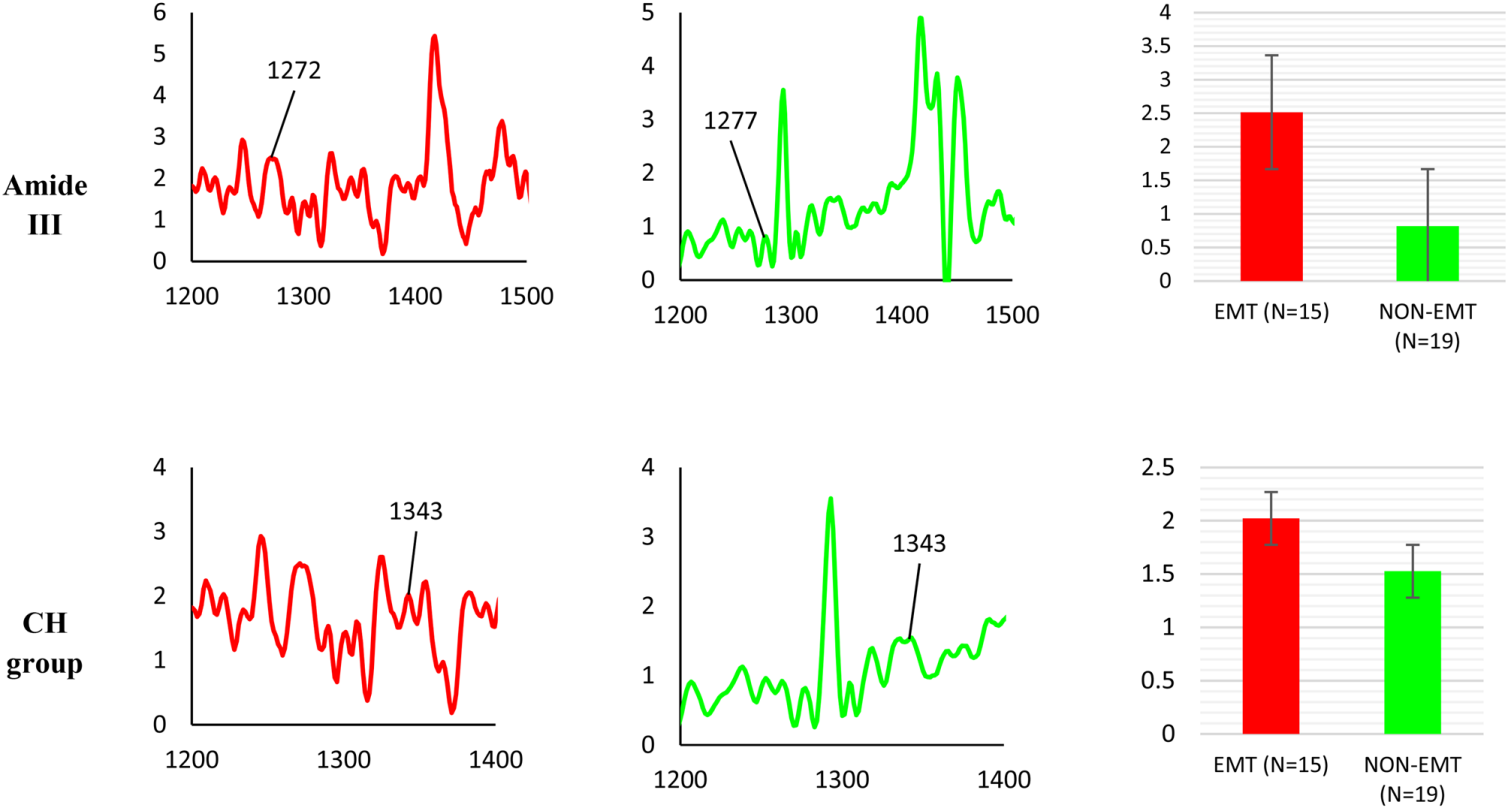
Summary of protein vibrational bands observed for waxed samples (Group 2) of EMT and non-EMT breast cancer tissue. For each case, the x-axis represents the wavenumber while the y-axis represents the intensity. The colour black represents the non-lesional tissues, while red represents EMT tissues and green represents non-EMT tissues. Comparison of the relative intensity of protein Raman bands for waxed samples of EMT and non-EMT breast tissue us depicted in the bar charts. Each column represents the average of spectra for each type of tissue. The value of N represents the number of Raman spectra acquired for each sample, shown within the brackets. The error bar represents standard deviation. Note: the x-axis represents the tissue type and y-axis is Raman intensity. No statistically significant differences between the groups are reported by the t-test.

In the waxed samples, Amide III and CH group proteins were detected in both EMT and non-EMT tissues, with Amide III found at 1272 cm^−1^ in EMT tissues and 1277 cm^−1^ in non-EMT tissues. The CH group of protein are observed at 1343 cm^−1^ in both EMT and non-EMT tissues. Figure 7 presents mean Raman spectra for peaks attributed to proteins in both EMT and non-EMT breast tissues samples. Comparison is made of the intensities of the protein bands for all samples (Fig. 7). There is some evidence (see below) of associations between proteins and cancer^63–66^, in regard to present interest playing an important role in the EMT process, including overexpression of certain proteins. Among these, in cells that have been observed to be in progression towards invasive forms of breast cancer are: Vimentin, N-cadherin, snail family zinc finger 1 (SNAIL), snail family zinc finger 2 (SLUG), twist family bHLH transcription factor 1 (TWIST), zinc finger E-box binding homeobox 1 (ZEB1) and zinc finger E-box binding homeobox 2 (ZEB2)^67,68^. It has been reported that high protein levels are linked to the division of cells, migration and gain cell proliferation in tumours^61^. Previous studies by others, using Raman spectroscopy on breast cancer tissues (but not on EMT specifically), have shown cancerous breast tissues contain more protein^62,69^. The protein bands found in this study are dominated by the amide group.

The increased intensities of protein bands in Raman spectra are a result of increased proliferation in the EMT tissues, the Snail family having been shown to be involved in proliferation control during EMT processes^70^. The latter is described by a distinct pathway that is explored in direct association with cancer progression (i.e. to trigger EMT-associated process), involving the protein tyrosine phosphatase (PTP) Pez^71^. The protein’s phosphorylation on tyrosine residues via tyrosine kinases and the reversible tyrosine phosphorylation catalysed by PTP regulating various cellular functions from cell proliferation to differentiation. Study made by Wadham’s group identified that the PTP Pez is an intracellular PTP localised to the adherents junctions (AJ) in endothelial and epithelial cells^71–73^.

Present work found that the intensity of CH group protein in the dewaxed samples is greater in EMT tissues compared to that of non-lesional and non-EMT tissues, by 18% and 49%, respectively. Meanwhile, in the waxed samples, the intensity of CH group protein is found to be 28% greater in EMT tissues compared to non-EMT tissues. The CH group protein results obtained herein are similar to that found by previous researcher; Hu et al. 2013^74^. Li et al. 2017^62^ observed Amide III in cancerous tissue and close to absent in normal tissue, where present results has found that the intensity of Amide III in EMT tissues is greater to that for non-lesional tissues and non-EMT tissues, by 41% and 18% respectively. For the case of waxed samples, the intensity of Amide III is 102% greater in the EMT tissues compared to the non-EMT tissues.

#### Nucleic acid

**Figure 8:**
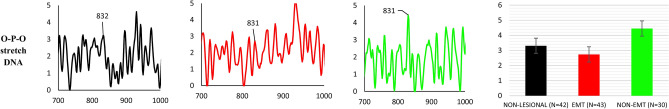
Summary of nucleic acid vibrational bands observed for dewaxed samples (Group 1) in non-lesional, EMT and non-EMT breast cancer tissue. For each case, the x-axis represents the wavenumber while the y-axis represents the intensity. The colour black represents the non-lesional tissues, while red represents EMT tissues and green represents non-EMT tissues. Comparison of the relative intensity of nucleic acid Raman bands for dewaxed samples in non-lesional, EMT and non-EMT breast cancer tissue is depicted in the bar chart. Each column represents the average of spectra for each type of tissue. The value of N represents the number of Raman spectra acquired for each sample, shown within the brackets. The error bar represents standard deviation. Note: the x-axis represents the tissue type and y-axis is Raman intensity. No statistically significant differences between the groups are reported by the ANOVA test.

**Figure 9:**
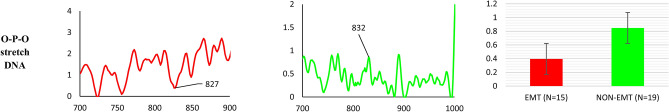
The nucleic acid vibrational bands of O–P–O stretch observed for waxed samples (Group 2) in non-EMT breast cancer tissue. Each column represents average of spectra for each type of tissue. The value of N represents the number of Raman spectra acquired for each sample, shown within the brackets. The error bar represents standard deviation. The x-axis represents the wavenumber while the y-axis represents the intensity. No statistically significant differences between the groups are reported by the t-test.

Raman spectra of nucleic acid bands were found in FFPE dewaxed tissue non-lesional, EMT and non-EMT samples. The peak assignments are shown in Fig. 8. The relative intensities of nucleic acid band in non-lesional, EMT and non-EMT tissues were compared, as shown in Fig. 8. The O–P–O stretch of DNA was found in non-lesional tissue at 832 cm^−1^ and at 831 cm^−1^ in both EMT and non-EMT breast cancer tissues. For the case of FFPE waxed tissue samples, Raman spectra of the O–P–O stretch DNA bands are found in both EMT and non-EMT tissues, at 827 cm^−1^ and 832 cm^−1^ respectively, as shown in Fig. 9. The comparison of relative intensities for both EMT tissues are also included in Fig. 9.

Nucleic acids are basic molecules within which all the information needed to control and build cells are stored. Therefore, the nucleic acids may be excellent biomarkers for screening of breast cancer. In the study by Li et al. on discrimination of breast cancer tissues from normal tissues, the O–P–O stretch DNA signal was observed to be attenuated in cancer tissues^75^. In the present study, an attenuated nucleic acid signal is also observed in EMT tissues, reflecting the changing configurations and components of nucleic acids during EMT processes in cancer progression. For the dewaxed samples, the intensity of O–P–O stretch DNA is found to be 19% greater in non-lesional tissues compared to EMT breast cancer tissues and 48% higher in non-EMT tissues compared to that EMT tissues. In the waxed samples, the O–P–O stretch DNA of non-EMT tissues is 73% higher compared to the EMT tissues.

**Figure 10:**
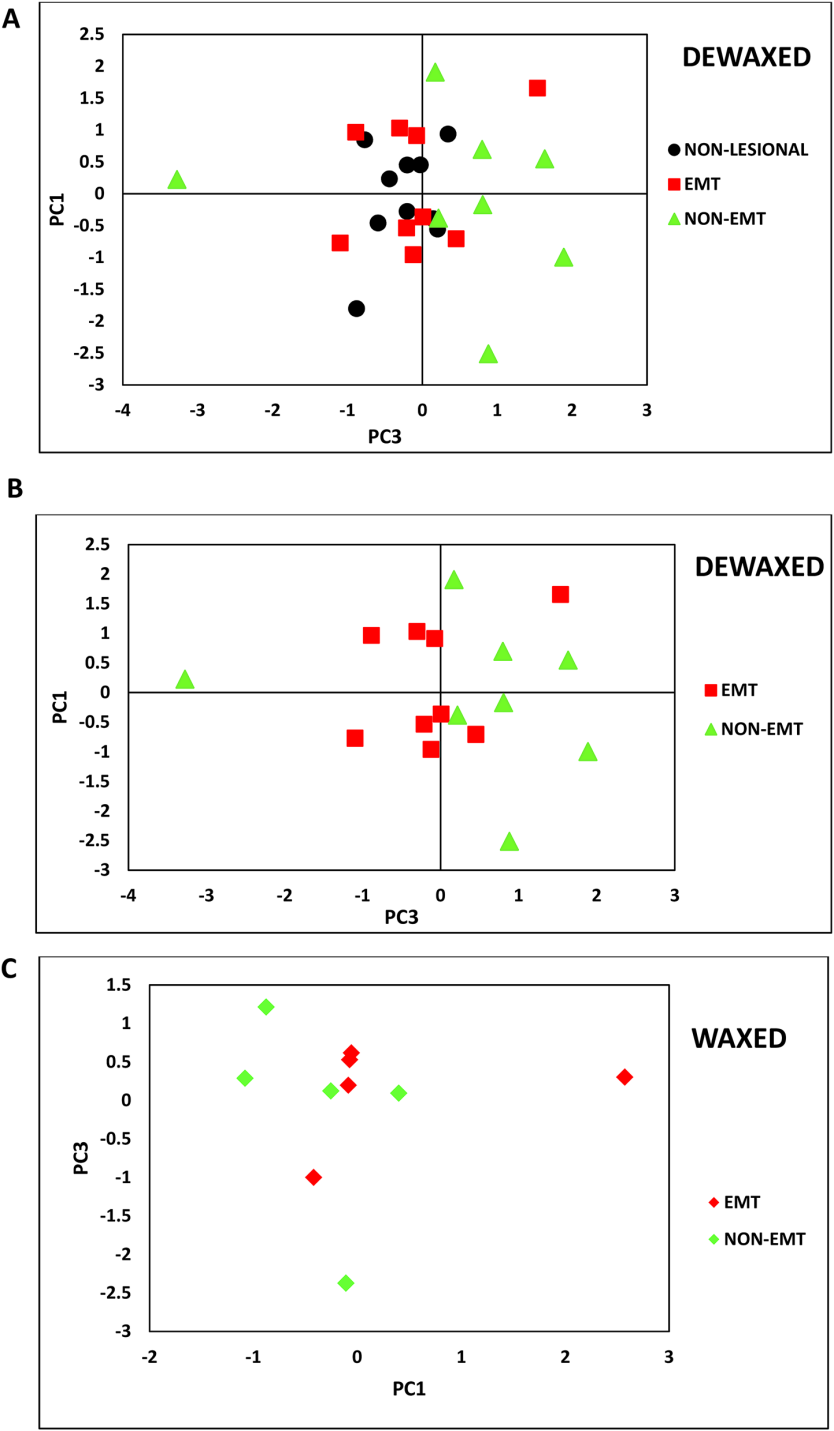
PCA plots of all studied samples; non-lesional, EMT and non-EMT breast tissues of both dewaxed (**A** and **B**) and waxed (**C**) samples.

The general trends in the data from these measurements were studied using PCA (Fig. 10). Two analyses of PCA were performed. The first PCA was performed on the dewaxed samples and the second PCA was performed on the waxed samples The method used for the analyses are described below:

1. Dewaxed samples:
2. Non-lesional (N = 10)
3. EMT (N = 9)
4. Non-EMT (N = 8)

4. Waxed samples
1. EMT (N = 5)
2. Non-EMT (N = 5)

For the dewaxed samples, the data obtained from PCA are shown in Fig. 10A and B. For Fig. 10A, the PCA results show that majority of the non-lesional samples are clustered in the negative area around the PC3 axis with values of between 0.00 to  − 1.00. Figure 10B which is part of Fig. 10A, focuses on the EMT and non-EMT samples. From the figure, it shows that the majority of EMT samples are clustered in the negative area from 0.00 to  − 1.00 along the PC3 axis while the majority of non-EMT samples are clustered in the positive area from 0.00 to 2.00 along the PC3 axis. For the waxed samples, the score plots of the waxed samples in Fig. 10C show the presence of two groups with one outlier from the EMT group. PC1 and PC3 account to 62.6% of the total variance.

The ANOVA results for intensity show that significant differences exist, and the Tukey’s multiple comparisons test revealed statistical significance while considering the EMT—non-EMT pair (*p*-value < 10^−4^) as well EMT—non-lesional pair (*p*-value < 10^−7^). On the other hand, the ANOVA test reports that there is a significant difference between waxed and dewaxed samples (*p*-value <10^−16^) when intensity is of concern.

Finally, it is important to examine the biochemical and medical biochemistry projections that may have traction in considering the present combination of Raman spectroscopy and histology findings. In respect of cell transformation in the breast, present work shows highly increased phospholipid content with saturated C–C stretching in stromal mesenchymal cells, confirming fatty acid accumulation due to limited fatty acid breakdown, being not only a key characteristic of tumour cells with rapid growth and poor prognosis but also in stromal cells to support their growth. Attention is drawn to potential links to several original contributions within which deupleting metabolic markers have been demonstrated clinically. These involve lung^76^, rare childhood cancers^77^, renal cell cancers^78^ and colorectal cancers^79^, until now lacking evidence in breast cancer. The growing field of what is referred to as deutenomics has consistently shown the increased significance of deuterium depletion via natural cellular ketogenic substrate oxidation. The underlying medical biochemistry mechanisms, described by Boros et al.^80,81^, have considered how defective mitochondria with diminished low deuterium ketogenic fatty acid substrate oxidation can hamper recycling of deuterium depleted metabolic water. This is seen to be performed by tricarboxylic acid cycle (TCA cycle) hydratase reactions^82^. Such mechanism could preserve normal epithelial cellular mesenchymal phenotype in breast to prevent or reverse cancer formation.

Notwithstanding the above, we acknowledge that breast cancer can be very heterogenous in terms of histological types and molecular classification. In order to minimise that, we have only included invasive ductal carcinoma (the most common histological type) in this study, and excluded lobular carcinoma, sarcomas and pre-invasive cancers such as in-situ carcinomas. Although data on the molecular predictive classification of the cancers are presented, no attempt is made here to correlate it with EMT status as the current study is not designed for that. That aspect is being addressed in a larger concurrent study.

We recognised that the EMT process itself is dynamic and manifests in a spectrum of morphological and biological expressions. As our study uses only formalin-fixed paraffin-embedded human tissues, many technologies cannot be reliably applied to such processed tissues to explore the spectrum of changes e.g. genomic and next generation sequencing techniques and many pathway regulators such as EMT-TFs. For FFPE tissues, the most reliable methodology to study biomarkers is immunohistochemistry, which has the advantage of direct visualisation of cancer cells. Unfortunately, the incorporation of a broader range of IHC-feasible biomarkers such as b-catenin and p120 catenin was beyond the scope of the study grant. The combination of E-cadherin and Vimentin was the most practical within our range for exploring EMT status as these were well-tested biomarkers with reliable controls in our laboratory. The varying expressions (through semiquantitative scoring system based on percentage and intensity) of E-cadherin and Vimentin allowed recognition of the complete EMT state versus the intermediate states of EMT-C and EMT-V. Also, by selecting only invasive ductal carcinomas, we have excluded the stages of disruption of apical-basal polarity (seen in in-situ/preinvasive carcinomas) and breach of the basement membrane (seen in early invasive carcinomas) of the early EMT process. However, in this study rigorous analysis of waxed against dewaxed samples in comparison to control samples has not been undertaken prior to ensure the changes in the analysis parameters or alter interpretability of the pre-processing data. These various steps cannot be explored as only FFPE archived material is available for study.

Since EMT is a plastic and heterogenous state, it is expected that different studies may demonstrate differing prevalences of the complete and intermediate types of EMT. Also, depending on the number and types of epithelial or mesenchymal markers utilised, the categorisation of various hybrid states of EMT may vary in different studies. Our study attempted to conform to the recent nomenclature guidelines^7^ within the limitations of the biomarkers we utilised. Although our study was not designed to determine prevalence, we observed that of 12 EMT positive cases in our study, 3 (25%) were EMT-C and 2 (16.7%) were EMT-CV. The majority (7 cases) were EMT-V (58.3%). This distribution is comparable to a recent tissue microarray immunohistochemical study by Jørgensen et al. (2020)^83^ where breast cancers which expressed both loss of epithelial and gain of mesenchymal markers (classified as “mesenchymal” in their study and equivalent to EMT-CV in our study) were almost equal in number as those that had absence of both epithelial and mesenchymal markers (classified as “negative” in their study and equivalent to EMT-C in our study). Their study also showed that tumours with retention of epithelial markers but gain of mesenchymal markers (classified as “partial’ in their study and equivalent to EMT-V in our study) was the larger majority. Another recent immunohistochemistry study^84^ on EMT in breast cancer showed E-cadherin-negative, vimentin-negative tumours (equivalent of EMT-C) to exceed E-cadherin-negative, vimentin-positive (equivalent of EMT-CV) and E-cadherin-positive, vimentin-positive (equivalent of EMT-V) tumours in prevalence.

## Conclusion

Inspection of biological tissue via Raman spectroscopy can yield a wealth of information regarding molecular bonds, potentially leading to the discovery and quantification of new intrinsic biomarkers associated with tissue classification. Particular instances may distinguish between ‘diseased’ and ‘normal’ tissue or between different stages of disease progression. In a laboratory setting, we demonstrated the Raman spectral analysis applied in unison with a combination of multivariate statistical techniques (PCA, ICA and NNLS) to discriminate cancer phenotypes, in particular EMT and non-EMT of breast tissue embedded in paraffin wax. The tissue samples have been investigated in dewaxed and waxed conditions. It was found possible to extract information concerning the relative quantities of various profiles of lipid, protein and nucleic acid composition of the breast tissues investigated herein. PCA indicated that the tissues of the EMT phenotype are potentially distinguishable, producing a spectral signature of the proclivity to mesenchymal transition, related to highly aggressive and metastatic spread. The ANOVA test followed by the Tukey’s multiple comparison test showed that significant differences exist among non-lesional, EMT and non-EMT samples based on Raman intensity. Human breast tissues were used, to our knowledge being the first study tracking the molecular content of cancer cells in human tissue during the EMT process. The findings are summarised as follows:

1. For dewaxed samples, we found that CH lipid bands in non-lesional tissues are 42% and 21% greater in intensity in comparison to that EMT and non-EMT tissues, respectively. For the signal of waxed tissue samples, the CH group of lipid is 37% greater in non-EMT tissues compared to that of EMT tissues.
2. Phospholipid bands greater in intensity by 60% and 110% were found in EMT tissue compared to non-lesional and non-EMT respectively. For the waxed samples, the phospholipid was found in both EMT and non-EMT tissues with slightly higher intensity in EMT tissues by 2% compared to that non-EMT tissues. Accordingly, the increase in lipid is associated with the process of EMT in breast cancer.
3. The greatest intensity of lipid C–C stretching was found in the EMT tissues; 109% higher compared to that non-EMT tissues and 37% higher compared to that of non-lesional tissue in the dewaxed samples. In the waxed tissues, C–C stretching of lipid was found to be greater in intensity by 21% in EMT tissues compared to that in non-EMT tissues.
4. In the dewaxed samples, the intensity of CH group protein was found to be 18% and 49% greater in EMT tissues compared to that of non-lesional and non-EMT tissues, respectively. In the waxed samples, the intensity of CH group protein was found to be 28% greater in EMT tissues compared to non-EMT tissues.
5. In the dewaxed samples, the intensity of Amide III in EMT tissues has been found to be 41% greater compared to non-lesional tissues and 18% greater in non-EMT tissues. In the waxed samples, the intensity of Amide III has been found to be 102% greater in the EMT tissues compared to the non-EMT tissues
6. Attenuated levels of O–P–O stretch DNA have been observed in EMT breast tissues, reflecting the changing configurations and components of nucleic acids during the EMT processes in cancer progression. For the dewaxed samples, the intensity of O–P–O stretch DNA is found to be 19% greater in non-lesional tissues compared to EMT breast cancer tissues and 48% higher in non-EMT tissues compared to that EMT tissues. In the case of waxed samples, the O–P–O stretch DNA of non-EMT tissues has been observed to be 73% higher compared to the EMT tissues.
7. Raman spectroscopy, supported by multivariate analysis (PCA, ICA and NNLS), is found to be a reliable instrument in assessing metabolic changes in EMT breast cancer tissues.
8. In terms of intensity, the results from ANOVA affirm that dewaxed and waxed samples differ significantly.

Present results point to a particularly important aspect concerning the utility of the Raman techniques that have been applied herein, namely the possibility of undertaking evaluation of the enormous archival tissue material available in clinical diagnostic laboratories. In this, one can see potential utility in further assisting in stratification of cells in terms of the metastasis initiation process phenotype.
